# Supplementary material for: Multi‐Space Excitation as an Alternative to the Landauer Picture for Nonequilibrium Quantum Transport
Source: Adv Sci (Weinh). 2020 Jun 30;7(16):2001038. doi: 10.1002/advs.202001038 (PMC7435256; doi:10.1002/advs.202001038)
Supplement: Supplementary file 1 — Supporting Information [file ADVS-7-2001038-s001.pdf]

Copyright WILEY-VCH Verlag GmbH & Co. KGaA, 69469 Weinheim, Germany, 2018.

Supporting Information

Multi-space excitation as an alternative to the Landauer picture for no-equilibrium quantum transport

*Juho Lee, Han Seul Kim, and Yong-Hoon Kim**

The numerical convergence of transmission spectra with respect to the size of electrodes

Within the multi-space constrained-search density functional theory (MS-DFT) approach, as discussed in the section **Formulation of MS-DFT**, finite-sized metallic electrodes are adopted as an alternative to the semi-infinite electrodes within the DFT-based non-equilibrium Green's function (NEGF) framework. Thus, to perform MS-DFT calculations, one might question bigger-sized electrodes might be required compared with DFT-NEGF calculations. To systematically determine the critical size of the electrodes required in MS-DFT calculations compared with DFT-NEGF calculations,[1] we calculated the transmission spectra of a benzenedithiolate (BDT) sandwiched between 3 × 3 Au(111) electrodes at V_b = 0.8 V by varying the number of Au atomic layers within each electrode from four layers (4L) to six layers (6L) and to eight layers (8L). In all calculations, the outermost three atomic layers were treated as the reservoir regions in both MS-DFT and DFT-NEGF calculations (see **Experimental Section** for details).

As shown in **Figure S1a**, we observe that within DFT-NEGF the transmission spectra

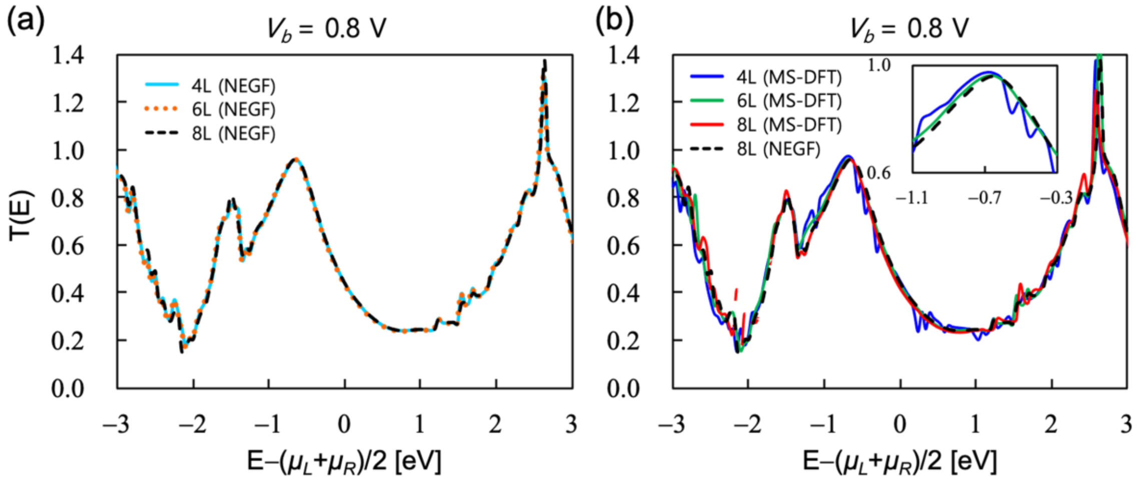

Figure S1. The numerical convergence of the transmission spectra calculated for a BDT junction under the bias voltage V_b = 0.8 V. In both (a) DFT-NEGF and (b) MS-DFT calculations, we tested the four-layer (4L), six-layer (6L), and eight-layer (8L) Au electrode cases. (Inset) Zoomed-in MS-DFT transmission peaks corresponding to the BDT highest occupied molecular orbital level.

are converged to a satisfactory level already at the 4L case (one surface Au layer as the lead region).[1] This immediate convergence might represent the advantage of introducing self-energy matrices within the grand-canonical Landauer picture at the cost of performing separate bulk DFT calculations and introducing the ambiguity of combining three different DFT calculation results.

Within the MS-DFT approach, as shown in **Figure S1b**, we also observe that the numerical convergence is systematically achieved and the full convergence is reached at the Au 6L level (green solid line). We can thus conclude that adding a few atomic layers (in the current example, 2 Au atomic layers or 18 Au atoms per electrode) within the MS-DFT calculation can achieve a satisfactory level of agreement with the standard DFT-NEGF calculation. Note also that the requirement of a larger electrode size does not translate into a higher computational cost because often MS-DFT converges faster than DFT-NEGF. Accordingly, given the many theoretical advantages described in the main text, we claim that MS-DFT represents an outstanding option for non-equilibrium quantum transport calculations.

References

[1] Y.-H. Kim, J. Kor. Phys. Soc. 2008, 52, 1181.
